# Quantification of Barley Contaminants in Gluten-Free Oats by Four Gluten ELISA Kits

**DOI:** 10.1021/acs.jafc.1c07715

**Published:** 2022-02-14

**Authors:** Xin Huang, Hanna Ahola, Matthew Daly, Chiara Nitride, EN Clare Mills, Tuula Sontag-Strohm

**Affiliations:** † Department of Food and Nutrition, Faculty of Agriculture and Forestry, University of Helsinki, FI-00014 Helsinki, Finland; ‡ Manchester Institute of Biotechnology, Division of Infection, Immunity and Respiratory Medicine, Faculty of Biology, Medicine and Health, University of Manchester, Manchester M17DN, U.K.; § Department of Agricultural Sciences, University of Naples Federico II, 80055 Portici, Italy

**Keywords:** codex, celiac disease, R5, G12, overestimation, calibrator, reference material

## Abstract

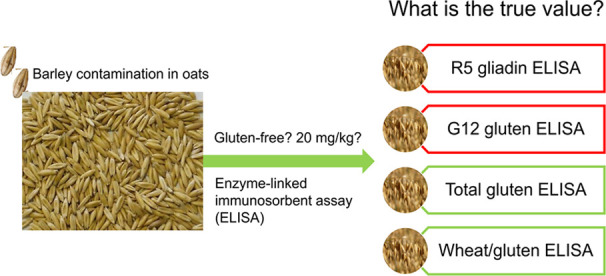

Pure oats are generally accepted to be safe for most celiac patients, and consumption of oats provides advantageous dietary fibers. However, oats can be contaminated by gluten proteins from wheat, barley, and/or rye. The analytical challenge lies in the reliability of the quantification method and how to maintain the contamination level under a gluten-free food threshold of 20 mg/kg. In this study, we investigated barley-spiked oat flour samples at four levels using four gluten ELISA kits. The largest recovery variance was with the R5 kit that gave 5–6 times overestimation; the G12 kit cross-reacted with oat proteins and gave 4–5 times overestimation at all spiked levels. The Total Gluten and Morinaga kits gave satisfactory recoveries. Total barley hordeins were isolated and characterized to be used as a common calibrator in all four kits aiming at harmonizing the results and to test the kits’ performance. Immunoblotting of total hordein isolate revealed that Total Gluten and Morinaga antibodies provided an overall detection, while R5 and G12 antibodies recognized specific hordein groups leading to a larger difference when wheat and barley were used as the calibrant. Calibration with total hordein isolate corrected the overestimation problem and decreased the variability between the four gluten kits.

## 1 Introduction

Celiac disease is an autoimmune disease triggered by ingestion of gluten proteins causing gastrointestinal disorders in genetically predisposed individuals.^1^ Gluten proteins are seed storage proteins in cereals and are referred to as gliadins and glutenins in wheat, hordeins in barley, and secalins in rye. They are insoluble in water and salt solution, and because of their high content in Pro and Gln residues, gluten proteins are also called prolamins.^2^ Currently, no cure exists for celiac disease, and therefore, patients must follow a strict gluten-free diet. According to Codex Alimentarius 118-1979,^3^ dietary food products can be claimed as gluten-free when the tested gluten level is below 20 mg of gluten per kg of food (mg/kg). The standard has classified the enzyme-linked immunosorbent assay (ELISA) based on the monoclonal antibody (mAb) R5 as the type I method for gluten quantification in foods. The mAb R5-based sandwich-type ELISA has been found to be a useful quantitative method for gluten contamination in specifically corn and corn-based foods (AACC Method 38-50.01)^4^ and also has been endorsed by AOAC Official Methods Board for determination of gluten in foods containing wheat, barley, and rye (AOAC OMA 2012.01).^5^ Except R5-based methods, commercial ELISA kits based on other antibodies have been developed. The mAb G12 sandwich ELISA is suitable for gluten in rice flour and rice-based products (AACC Method 38-52.01 and AOAC OMA 2014.03).^6,7^ The FDA recognized the “Morinaga method” based on a polyclonal antibody alongside the R5 ELISA for gluten detection in foods (FDA 78 FR 47154, 2013),^8^ although the Morinaga method was, in fact, intended for wheat allergen protein detection, including water-soluble proteins and prolamins.

However, commercial gluten ELISA kits perform differently, having large variations within and between kits. The challenges in gluten detection and comparison of ELISA have been critically discussed, and three main issues have been identified in the ELISA procedure: sample extraction, antibody detection, and calibration processes.^9−12^ First, the extraction process is challenging, as the gluten proteins are heterogeneous and complex in structure, have solubility differences, and are highly affected by the processing history of the food. Second, the detection process varies due to the differences in the chosen antibody’s specificity and sensitivity. Each antibody used in gluten detection is raised against a certain gluten protein or gluten protein type; for example, the mAb R5 was raised against rye secalins,^13^ mAb G12 against 33-mer peptide originated from α2-gliadins,^14^ mAb Skerritt against ω-gliadin,^15^ and Morinaga polyclonal antibodies (pAb) against wheat proteins. Third, currently there is no certified reference material for calibration and it is not straightforward to find an appropriate and representative calibrant for all cereals and all types of ELISA systems.

A specific situation has drawn more attention recently to gluten-free oats. Despite the debate of safety of oats for celiac patients, the current evidence suggested that most celiac patients can tolerate uncontaminated pure oats.^16−19^ Uniquely, consumption of oats beta-glucan has gained health claims related to cholesterol, blood sugar regulation, and bowel movement.^20−23^ Other bioactive compounds, oat avenanthramides^24^ and oat polar lipids,^25^ have gradually gained attention due to their positive benefits. However, oats are often contaminated by wheat, barley, and/or rye, of which barley is the predominant contaminant.^26^ Quantification of barley contamination by R5 ELISA with gliadin standards^27^ led to severe unacceptable overestimation.^28−31^ One reason was that the high binding affinity of R5 antibody against barley C-hordein was observed due to its high number of QQPFP epitope repeats, and another reason was that the composition of barley hordeins differs from the calibrant, which is wheat gliadin. In addition, a conversion factor of 2 from prolamin content to total gluten content is not valid for barley, or not even always for bread wheat.^32,33^ The calibration using a barley total hordein standard could correct the overestimation in R5 ELISA^28,29^ when testing for barley contamination. A C-hordein isolate (40% mixed with an inert protein, which does not react with R5) has been proposed for total barley hordein calibration,^30^ and this calibrant (10% mixed with an inert protein) has been applied in wheat gluten calibration in R5 ELISA.^33^ However, the origin of the gluten contaminant is usually unknown. An ELISA kit, Total Gluten, with multiple antibodies was recently developed to solve this problem that the total gluten contents of wheat, barley, and rye are detected in an oat matrix.^34^

The aim of this study was to examine the efficiency of four gluten ELISA kits with barley contaminants in oat flour, including R5, G12, Total Gluten, and Morinaga. We identified the hordein recognition in these four ELISA kits and a total hordein isolate was prepared and served as a common calibrant in order to harmonize the ELISA results across the four kits and to test their proficiency at different spiking levels.

## 2 Materials and Methods

### 2.1 Materials

All reagents were of analytical grade. Barley *Hordeum vulgare* L. seeds cv. Brage were from Boreal Plant Breeding (Jokioinen, Finland), and oat *Avena sativa* seeds cv. Peppi and cv. Avetron were from Kinnusen Mylly Oy (Utajärvi, Finland). Barley cv. Brage is one of the most common cultivars for feed and malting in Finland; its C-hordein proportion was representative and was previously studied in a barley cultivar collection.^30^

### 2.2 Preparation of Total Hordein Isolate

Total hordein fraction from barley cv. Brage was isolated and used as a common calibrator in all ELISA kits. The isolation procedure was lightly modified from a previous wheat gluten isolation process.^33^ The seeds were milled with a Retsch ZM 200 (Haan, Germany) to a particle size 0.5 mm screen followed by a defatting step with defatting solution (methanol/diethyl ether 1:1, v/v) at a ratio of 1:5 (w/v) for 60 min at ambient temperature with constant magnetic stirring. After filtration, the defatted flour was dried overnight and then washed using 67 mM phosphate buffer (pH 7.4) with 0.4 M NaCl for 30 min at a ratio of 1:5 (w/v) three times at ambient temperature to remove the albumins and globulins. Following centrifugation at 12,000 × *g* and a brief wash of the pellet with milliQ H_2_O, the total hordein was extracted using 50% (v/v) propan-1-ol with 60 mM dithiothreitol (DTT) at a ratio of 1:3 (w/v) at 60 °C for 30 min. Three consecutive total hordein extraction supernatants were combined and dialyzed against milliQ H_2_O with 0.01% acetic acid using a dialysis membrane with a 14 kDa cut-off (Sigma D9777). Following lyophilization, the hordein isolate was ground and homogenized in a mortar. The nitrogen content of the barley flour and total hordein isolate was determined by a Dumas combustion method (Leco 828, St.Joseph, MI) and multiplied by 5.7 to give the protein content (ICC Standard No.167).^35^

### 2.3 Characterization of the Total Hordein Isolate by SDS-PAGE, Immunoblotting, and RP-HPLC

The hordein isolate was dissolved in 4 × lithium dodecyl sulfate sample buffer with a 10 × sample reducing agent (NuPAGE, Thermo Fisher) and incubated at 90 °C for 10 min. An amount of 15 μg of total hordein isolate was separated on a 10% Bis-Tris gel with MOPS running buffer at 200 V for 50 min, and Mark 12 unstained standard and Novex Sharp Prestained standard served as protein molecular standards. The gel was stained by SimplyBlue Safestain and imaged by Alpha Imager HP (ProteinSimple, CA). To investigate the antibody recognition of the total hordein isolate, an immunoblot with the four kits’ “enzyme-conjugate” was conducted. The proteins were transferred to a polyvinylidene fluoride membrane using an XCell II Blot module system (Invitrogen, Thermo Fisher) at 30 V for 60 min in the presence of a transfer buffer containing 20% (v/v) methanol, 192 mM glycine, and 25 mM Tris-HCl pH 8.3. Following blocking the membrane with 5% (w/v) skim milk powder in 0.1 M phosphate buffered saline (pH 7.4) for 60 min at ambient temperature, the proteins were recognized by a diluted “enzyme-conjugate” (R5 1:11, G12 1:1.5, Total Gluten 1:4, and Morinaga 1:4), which was each kit’s own antibody conjugated with horseradish peroxidase. The membrane was stained by a chemiluminescent substrate (SuperSignal West Pico PLUS, ThermoFisher) and visualized with a ChemiDoc Touch Imaging system (Bio-Rad, Hercules, CA) by auto-exposure.

The hordein isolate solution was also separated using a C8 column (Discovery Bio Wide Pore 5 μm, 25 cm × 4.6 mm, Supelco Analytical, Sigma-Aldrich) with a matching guard column 2 cm × 4 mm connected to an Agilent Technologies 1200 HPLC series system with a diode array detector (Agilent, Santa Clara, CA). The separation gradient was from 24 to 56% (v/v) acetonitrile with 0.1% (v/v) trifluoroacetic acid (Buffer B) at 50 °C for 40 min at a flow rate of 1 mL/min followed by a clean-up with 90% Buffer B. According to the retention time, D-, C-, and B/γ-hordeins were separated and their composition was calculated based on the peak areas (ChemStation, Agilent Technologies).

To determine the total hordein content in total protein of the barley flour, the albumin + globulin fractions were removed with a buffer containing 67 mM phosphate buffer (pH 7.4) and 0.4 M NaCl three times and briefly rinsed with mQ water. The total hordein was extracted by 50% (v/v) propan-1-ol, 2 M urea, and 50 mM DTT in 100 mM Tris-HCl (pH 7.4) at 60 °C in a water bath with sonication for 15 min; four consecutive extractions from the same pellet were combined. Their protein concentration was determined by peak area using a bovine serume albumin standard (0–80 μg linear range). The total hordein content was 60.6% of total protein.

### 2.4 Preparation of Spiked Oats

The oat seeds (cv. Peppi and cv. Avetron) were manually cleaned with caution to ensure that there was no foreign seed contamination. After dehulling with an oat dehuller Rivakka (NIPERE Oy, Finland), oat seeds were milled with a Retsch ZM 200 (Haan, Germany) to a particle size of 0.5 mm. After confirming with R5 gliadin ELISA (R7001 R-Biopharm, Darmstadt, Germany) that the oat flour was <5 mg/kg gluten proteins, barley flour was step-wise spiked into the oat flour at four hordein levels at 80, 40, 16, and 4 mg/kg by sufficient manual mixing for approximately 5 min (Table SI1). The hordein content of barley flour protein was taken as 60.6% of total protein content. To examine the homogeneity of the spiking method, ten 1 g samples from the 16 mg/kg spiked oats were examined by R5 gliadin ELISA.

### 2.5 Four ELISA Kits

The four spiked samples were analyzed using four ELISA systems, including Ridascreen Gliadin (“**R5**”, R7001, R-Biopharm), AgraQuant gluten **G12** (Romer-Labs, Austria), Ridascreen **Total Gluten** (R7041, R-Biopharm, Germany), and Wheat/Gluten ELISA kit II (**Morinaga**, Japan). Table 1 summaries the details of each ELISA kit. Total hordein isolate served as a common external hordein standard for all kits. Three individual extractions of each spiking level were conducted, and three measurements of each extraction were performed. Two sets of kit standards and two sets of hordein external standards were also served. The recovery was calculated by dividing the calibrated gluten content results with the theoretical spiking hordein content.

**Table 1:** Characteristics of Four Gluten ELISA Kits

	R5	G12	Total Gluten	Morinaga
validation	AACC 38-50.01	AACC 38-52.01	AOAC SMPR	AOAC PTM 011804^49^
	AOAC OMA 2012.01	AOAC OMA 2014.03	2017.021	FDA 2013
	AOAC PTM 120601^48^			
sample size	1 g	1 g^a^	1 g	1 g
(I) sample to extraction buffer ratio (w/v)	(I) 1:40	(I) 1:40	(I) 1:40	(I) 1:19
(II) further dilution (v/v)	(II) 1:12.5	(II) 1:12.5	(II) 1:12.5	(II) 1:20
extraction conditions	(a) 50 °C, 40 min (patented cocktail solution)^b^	(a) 50 °C, 40 min (extraction buffer)	(a) 50 °C, 40 min (patented cocktail solution)^b^	extraction buffer including, 2-ME, SDS AT, overnight
	(b) AT^c^, 60 min (60% v/v ethanol)	(b) AT, 60 min (60% v/v ethanol)	(b) AT, 60 min (60% v/v ethanol)	
antibodies	mAb R5	mAb G12	mAb R5; mAb HMW GS; mAb LMW GSs	pAb wheat proteins
quantification range	LOD: 0.5 mg/kg gliadin	LOD: 2 mg/kg gluten	LOQ: 5 mg/kg gluten	LOD: 0.31 mg/kg wheat proteins
	LOQ: 2.5 mg/kg gliadin	LOQ: 4 mg/kg gluten		LOQ: 0.78 mg/kg wheat proteins
ELISA antibody binding steps: (1) first reaction; (2) washing; (3) second reaction; (4) washing; (5) color reaction	1:30 min	1:20 min	1:20 min	1:60 min
	2 & 4:3 times	2 & 4:5 times	2 & 4:3 times	2 & 4:6 times
	3:30 min	3:20 min	3:20 min	3:30 min
	5:30 min	5:20 min	5:10 min	5:20 min
calibrant	PWG gliadins	vital gluten	total gluten	wheat proteins^e^
calibration function^d^	cubic spline	dose–response curve, provided excel sheet	4-parameter function	4-parameter curve fit (cubic regression)
gluten calculation	gliadin × 2	as it is	as it is	wheat proteins × 0.85

a G12 kit instruction sample size 0.25 g, in actual test sample size was 1 g for all tests.

b Patented cocktail solution including 2-mercaptoethanol (2-ME), guanidine hydrochloride, phosphate buffered saline, WO 02/092633.

c AT, ambient temperature, 20–25 °C

d In this study, all ELISA calculations were conducted by agonist response-variable slope (four parameters) by GraphPad Prism 8.0.2.

e A mixture of 14 wheat cultivars and extracted based on the Japanese official guideline.^50^

### 2.6 Statistical Analysis

All ELISA results were analyzed by an ROUT method (robust regression and outlier removal) using coefficient *Q* = 1% to remove any outliers (GraphPad Prism 8.0.2). To investigate the ELISA kits’ proficiency at four spiking levels, *z*-scores of results interpolated using both kit calibration and total hordein calibration were calculated by using the formula *z* = (*x* – *X*)/*σ*, where *x* is the interpolated value, *X* is the theoretical spiking value (80, 40, 16, and 4 mg/kg), and σ is 25% of the theoretical spiking value.^10^ A value of 25% defines the maximum acceptable uncertainty and was used in food allergen proficiency tests (DLA 2019, ISO 13528, 2015).^36,37^ The Youden plots were made by plotting the value determined at each spiking level interpolated using both the kits’ own calibration and total hordein calibrations (MedCalc 18.9.1, Belgium).

## 3 Results

### 3.1 Characterization of the Hordein Isolate Serving as a Common Calibrant

**Figure 1:**
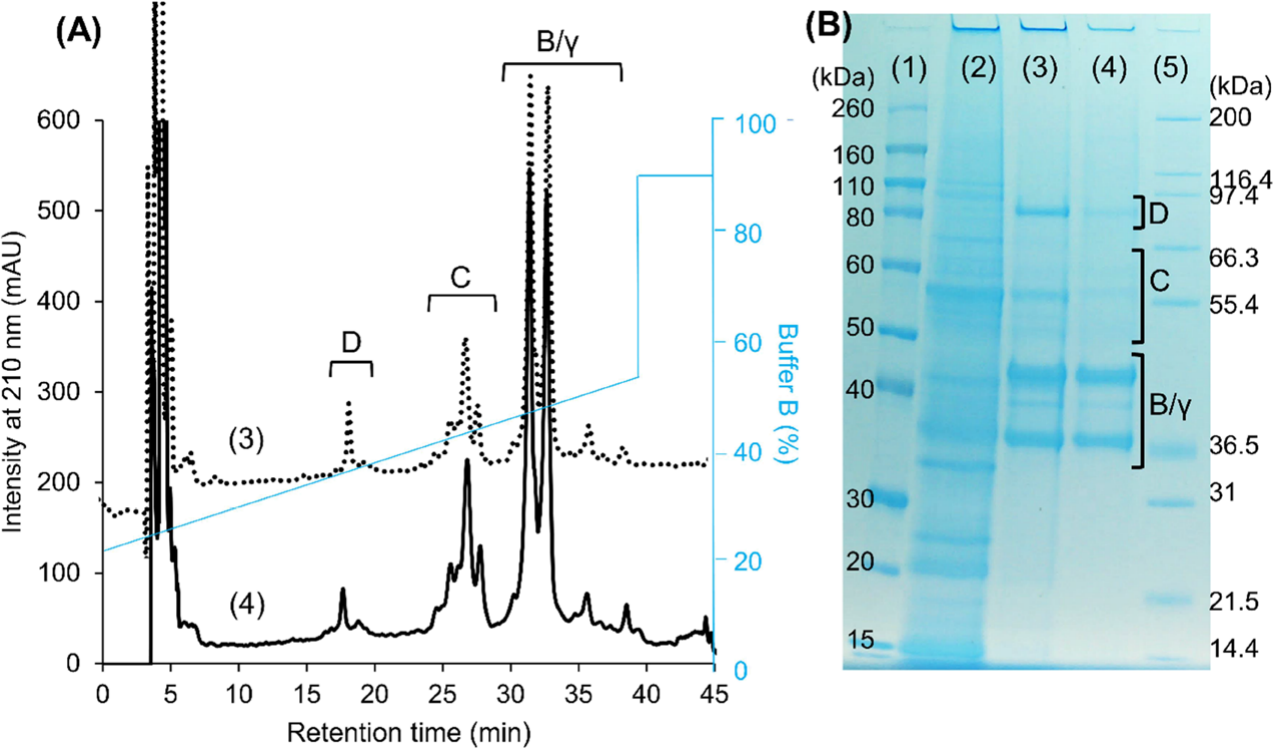
Characterization of the total hordein isolate by reverse-phase liquid chromatography (panel A) and SDS-PAGE (panel B). Lane 1, Novex Sharp Prestained protein standard. Lane 2, albumin + globulin extract of barley flour. Lane 3 and dotted line 3, total hordein extract from barley flour with albumin + globulin removed. Lane 4 and black line 4, total hordein isolate. Lane 5, Mark 12 unstained protein standard. The letters indicated the classification of hordeins, D, D-hordein; C, C-hordein; B/γ, B/γ-hordein.

Barley hordeins are divided into three groups based on their relative molecular mobility determined on the SDS-electrophoretic gel, including D-hordeins (70–90 kDa), C-hordeins (50–70 kDa), and B and γ-hordeins comigrating between 35 and 50 kDa.^38^ In reverse-phase separation based on protein hydrophobicity, the D-hordeins eluted first (15–20 min) followed by the C-hordein (18–28 min) and B and γ-hordeins in the chromatographic region of 28–40 min.^39^ The hordein composition of the barley flour, based on integrating peaks in RP-HPLC, was D-hordein 3.7 ± 1.0%, C-hordein 24.5 ± 2.1%, and B/γ-hordein 71.8 ± 2.2% of total hordein, while the composition in the hordein isolate was D-hordein 2.2 ± 1.0%, C-hordein 27.6 ± 1.9%, and B/γ-hordein 70.2 ± 2.0% of total hordein. The barley hordein isolate comprised all the hordein components as confirmed by SDS-PAGE and RP-HPLC analysis (Figure 1,BA). The sequential extraction removed most of the albumins and globulins prior to prolamin extraction based on comparison of the gel profile (Figure 1B lanes 2 and 3).

**Figure 2:**
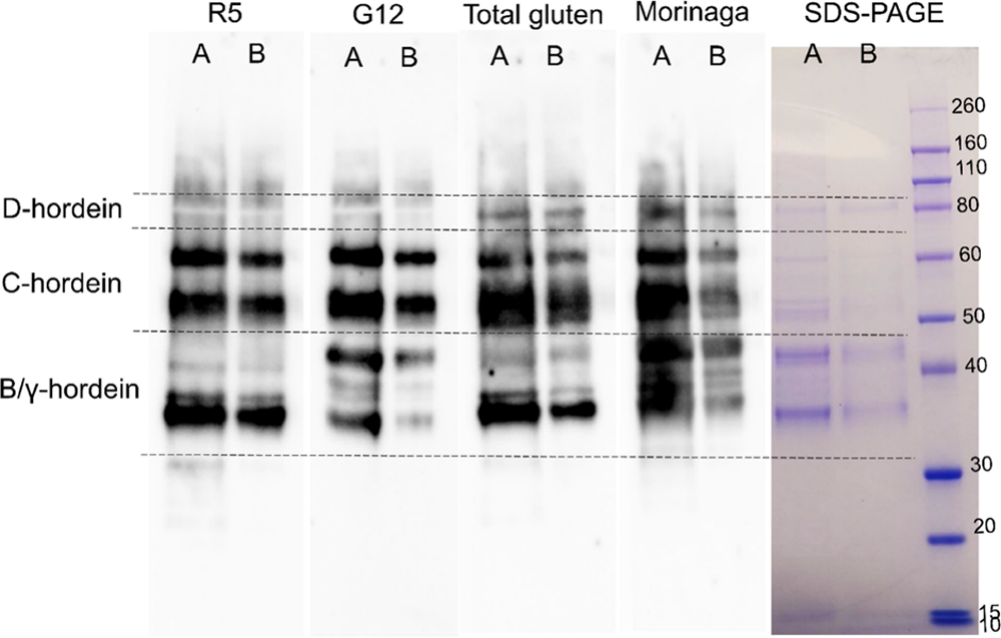
Immunoblot of total hordein isolate by an antibody-conjugate from four ELISA kits. Protein load to the gel lane A 2.5 μg and lane B 0.5 μg.

The specificity of different antibodies against barley hordeins was presented by immuoblotting. The R5 mAb mainly recognized C-hordeins and an ∼36 kDa band in the B/γ-hordein region but did not recognize D-hordeins (Figure 2). Similarly, the G12 mAb recognized C-hordeins but did not recognize D-hordeins. The G12 mAb recognized more proteins than the R5 mAb in the B/γ-hordein range at around 45 kDa. A combination of antibodies in the Total Gluten kit included the R5 antibody, a HMW-glutenin antibody, and LMW-glutenin antibodies. The recognition pattern of the Total Gluten kit antibody solution resembled that of the R5 where C-hordeins and B/γ-hordein were correspondingly recognized with slightly broader detection. Total Gluten kit antibodies also recognized D-hordeins. Morinaga wheat pAb recognized all groups of barley hordeins, especially in the B/γ-hordein region where a larger number of bands were recognized compared to the other three antibody systems.

**Figure 3:**
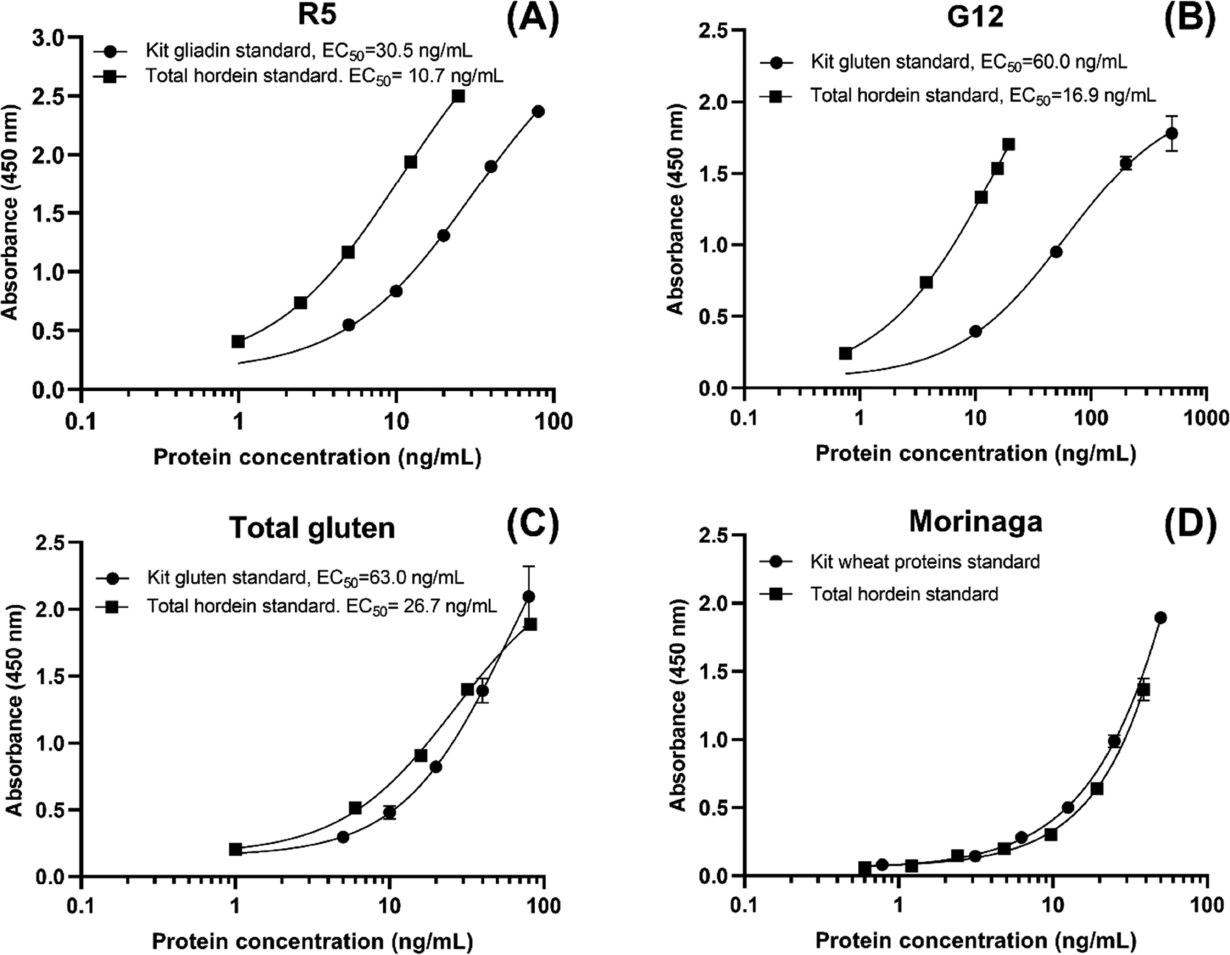
Comparison of each kit’s own calibration curve and total hordein calibration curve in four ELISA kits: (A) R5 gliadin vs total hordein ng/mL, (B) G12 gluten vs total hordein ng/mL, (C) Total Gluten total gluten vs total hordein, (D) Morinaga wheat proteins vs total hordein ng/mL. The total hordein was hordein isolate × protein content ng/mL. The curves were matched on a gravimetric basis. The curve was produced using agonist response-variable slope (four parameters) nonlinear fit, and the EC_50_ value was calculated from the best-fit curve values by GraphPad Prism 8.0.2 (San Diego, CA).

The comparison of the kits’ own standard calibration curve and external common standard total hordein calibration curve showed the difference of antibody reactivity against wheat gliadin/gluten proteins and barley hordeins (Figure 3). Their curve EC_50_ values revealed that in R5 and G12 kits, the total hordein isolate exhibited stronger binding with the antibodies than the wheat gliadin in the R5 kit and vital gluten in the G12 kit, while in Total Gluten, the difference of total wheat gluten and total barley hordein was less pronounced. It was not possible to determine the EC_50_ of the calibration curves in Morinaga because the top of the curve could not be identified. In fact, the Morinaga curves showed good fitness in the linear regression fit (*R*^2^ > 0.99). However, the difference between wheat proteins and total hordein was less than that for the other three kits, and the wheat proteins showed even slightly stronger binding than the total hordeins.

### 3.2 Barley in Oat Spiking Tests Using Four Gluten ELISAs

The homogeneity test of the 16 mg/kg barley-spiked oat sample with an R5 sandwich and gliadin standard resulted in CVs lower than 15%, showing that the spiking method was sufficient and satisfactory homogeneity of the spiked samples was achieved. For the nonspiked oats (0 mg/kg), the R5 and Total Gluten were below the quantification limit, and Morinaga showed a detectable gluten content (Table 2). The G12 kit showed cross reaction with oats and measured 9.8 ± 0.3 mg/kg. With the kits’ own calibrator, R5 and G12 both showed 3.7–6.1 times overestimation at four spiking levels, while Total Gluten and Morinaga kits showed satisfactory recovery (between 50 and 150%, corresponding to |*z*| < 2) when the spiking level was above the kit limit of quantification. Calibration with total hordein in all four kits resulted in satisfactory protein recovery in all spiking levels. The overestimation by R5 and G12 was corrected, while the Total gluten showed decreased recoveries and Morinaga showed increased protein recovery at all spiking levels. The recovery of the G12 kit was the lowest of four kits. As the spiking level increased, a slight decrease in protein recovery was observed in Morinaga kits.

**Table 2:** Calibration Results mg/kg Gluten Proteins from Barley-Spiked Oat Samples from Kit Standard Calibration and Total Hordein Calibration

	kit calibration ± SD (mg/kg)	kit calibration recovery (%)	total hordein calibration ± SD (mg/kg)	total hordein calibration recovery (%)
0 mg/kg				
R5	5.3 ± 0.3^a^		<LOQ^b^	
G12	9.8 ± 0.3		<LOQ^b^	
Total Gluten	<LOQ		<LOQ^b^	
Morinaga	3.1 ± 0.1		3.9 ± 0.1	
4 mg/kg				
R5	24.5 ± 3.8	613	<LOQ^b^	95
G12	17.2 ± 1.0	429	<LOQ^b^	54
Total Gluten	6.9 ± 0.7	173	3.7 ± 0.8	92
Morinaga	3.9 ± 1.0	98	4.9 ± 1.2	123
16 mg/kg				
R5	80.1 ± 6.8	501	11.6 ± 0.9	72
G12	84.9 ± 10.5	531	10.2 ± 1.0	64
Total Gluten	17.4 ± 1.0	109	10.2 ± 1.3	64
Morinaga	13.0 ± 1.6	82	15.7 ± 1.7	98
40 mg/kg				
R5	218.2 ± 25.0	545	32.8 ± 3.6	82
G12	210.0 ± 6.9	525	25.4 ± 0.7	63
Total Gluten	44.3 ± 4.0	111	30.5 ± 4.7	76
Morinaga	31.1 ± 4.0	78	37.7 ± 4.4	94
80 mg/kg				
R5	398.2 ± 19.0	498	57.5 ± 2.5	72
G12	298.6 ± 26.5	373	38.1 ± 2.9	48
Total Gluten	85.6 ± 11.7	107	51.8 ± 6.9	65
Morinaga	58.7 ± 1.7	73	71.5 ± 1.9	89

a One extraction replicate was <LOQ; the other two extraction replicates averaged 5.5 ± 0.1 mg/kg.

b The LOQ of hordein calibration was calculated from the lowest hordein standard concentration.

**Figure 4:**
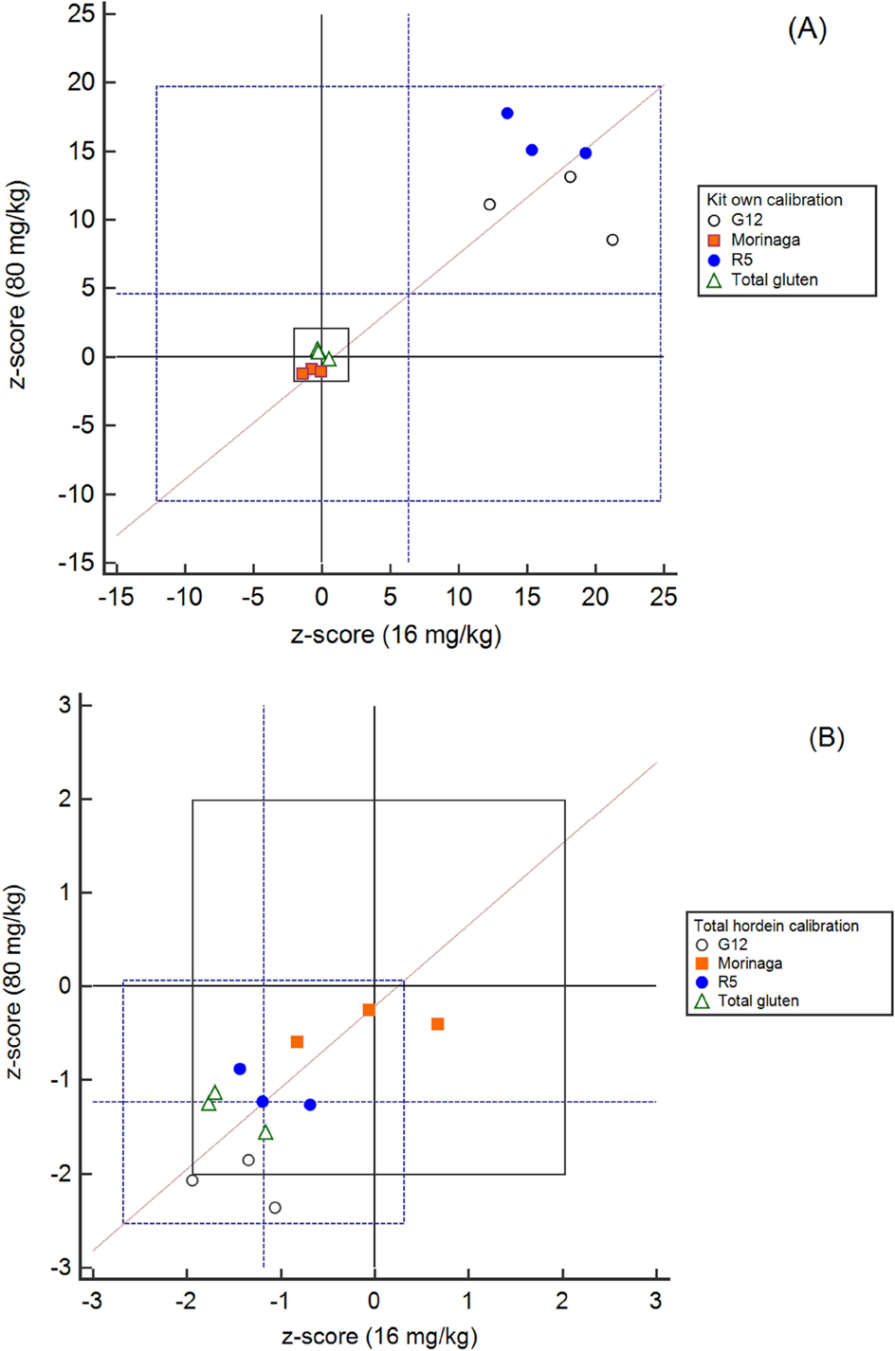
Youden plots of the *z*-score of two levels (16 mg/kg vs 80 mg/kg) of barley-spiked oats measured with four ELISA kits using the (A) kits’ own calibration and (B) total hordein isolate calibration. The black cross set at *z* = 0 was the theoretical spiking content, and the black rectangle was set to|*z*| ≤ 2, which was considered satisfactory in the proficiency assessment (ISO 13528). The blue cross was set at the mean value of all *z*-scores, and the blue rectangle was set at 2 times standard deviation of all *z*-scores. Three points from each ELISA kit indicated three individual extraction replicates.

The application of *z*-scores allows comparisons of the kits’ performances at the different spiking levels (Table SI 2). The Youden plot (Figure 4) is a visual presentation of *z*-scores at a low (16 mg/kg) spiking level and high (80 mg/kg) spiking level, which corresponded to be under the thresholds of the gluten-free (20 mg/kg) and low gluten content (100 mg/kg). Using the kits’ own calibration (Figure 4A), the four kits presented larger variation (*z*-score SD = 9.8 at 16 mg/kg, SD = 8.2 at 80 mg/kg) and resulted in the mean of *z*-scores >5, indicating unsatisfactory proficiency. All points were on the (+,+) quadrant of the plot alongside the 45 degree diagonal line, R5 and G12 ELISA kits had high *z*-scores >2 (13 to 20, 8 to 21, respectively) and thus were not satisfactory at any spiking level, showing a systematic error of overestimation. The Total Gluten and Morinaga kits exhibited satisfactory proficiency, where the |*z*|-scores ≤2, at both spiking levels. With the common calibrator total hordein, the mean of *z*-scores of the four kits was −1.04 at 16 mg/kg and 1.24 at 80 mg/mL within satisfactory proficiency, and the variance was lower with *z*-score SD = 0.68 at 16 mg/kg and SD = 0.69 at 80 mg/kg. Increasing the spiking level from low to high, the R5, Total Gluten, and Morinaga performed satisfactory (|*z*| < 2), while G12 showed decreased proficiency. Similar trends were observed in Youden plots of 4 vs 16, 16 vs 40, and 40 vs 80 mg/kg (Figure SI3). The extraction step of R5 and Total Gluten was the same; after common calibration with total hordein, the variance from antibody detection showed that R5 had a slightly better performance, even though not significant, than the Total Gluten (mean value of *z*-score at 16 mg/kg, R5 vs Total Gluten 1.11 vs 1.54; at 80 mg/kg, R5 vs Total Gluten 1.12 vs 1.31). This may be due to shorter binding time in the ELISA steps with the Total Gluten procedure.

## 4 Discussion

In this study, we investigated the performance of four gluten sandwich-type ELISA kits to quantify barley flour spiked in oat flour at four gluten levels. Each test kit includes its own extraction method, specific gluten antibody/antibodies, and a wheat-based calibrant. The R5 kit gave a 5–6 times overestimation at all four spiking levels. The G12 kit cross-reacts with oats giving about 4–5 times overestimation at all spiking levels. The Total Gluten kit and Morinaga kit resulted in satisfactory recoveries at all levels.

The overestimation problem with barley has been reported earlier with the R5 ELISA,^28−30^ and the same phenomenon was observed with rye contamination in oats.^31^ The reason was that the composition of wheat gluten and barley hordein largely differed and the gluten antibody specificity and sensitivity also varied. For the R5 antibody, the strongest reaction was with omega-type prolamins because of their high number of repeats of the QQPFP motif, for example, ω1,2-gliadins in wheat (Uniprot Accession D2KKB1, 18 repeats) and C-hordein in barley (Q41210, 17 repeats). The C-hordein proportion in barley is, however, higher than the proportion of ω1,2-gliadins in wheat. The ω1,2-gliadin proportion in a 27 wheat cultivar collection was 1.9–9.0% of total wheat protein or 4.6–11.0% of total gluten proteins.^33^ However, the C-hordein proportion in a 29 barley cultivar collection was always higher, constituting 9.9–19.8% of total barley proteins or 16.5–33.1% of total hordeins.^30^ A conversion factor of 2 to gluten content for barley only amplified the overestimation because the actual barley conversion was lower, ranging from 1.20 to 1.71.^32^ In fact, the conversion factor 2 is not even valid for bread wheat and has been found to range from 1.19 to 1.48^33^ or 1.32 to 1.66.^32^

For the G12 mAb, similar to the R5, the strongest reaction was with wheat ω1,2-gliadins and barley C-hordein (Figure 2).^40^ Extra bands in the B/γ-hordein region were recognized; this may due to G12 mAb showing a stronger recognition with polymeric B/γ-hordeins than monomeric B/γ-hordeins.^40^ The G12 mAb cross-reacted with nonspiked oats and reported 10 mg of gluten in kg of pure oats. Since the G12 mAb was raised against a 33-mer from wheat α-gliadins, a celiac model peptide, the G12 response with avenins in competitive ELISA has been correlated with T-cell-stimulating activity and can identify harmful oat cultivars.^41^ In another study, we have found that the sandwich G12 ELISA gave a 10–40 mg/kg in a collection of 26 oat cultivars,^42^ of which one cultivar (cv.Salo) was found safe in a clinical trial.^43^ After fractionation of avenin proteins, G12 was found to recognize the repetitive region of avenin PFVQ motifs.^42^ The G12 ELISA for oats was surrounded by great ambiguity due to the overestimation with barley hordeins and the cross reaction with oat avenins.

The Total Gluten kit introduced LMW-glutenin antibodies and an HMW-glutenin antibody. The LMW-glutenin antibodies did not recognize additional bands in the B/γ-hordein region, although they are homologous proteins, for example, LMW-glutenin (Uniprot Accession Q3W3V0) and B3-hordein (P06471) sharing 57.2% sequence identity after alignment (Uniprot BLAST tool). The specificity and the recognition epitopes of the LMW-glutenin antibodies were not revealed by the manufacturer. The D-hordein was recognized (Figure 2), possibly by the HMW-glutenin antibody, although the manufacturer claimed no detection of D-hordein in the Total Gluten kit. The D-hordein (Uniprot Accession Q40054) and HMW-glutenin subunit DY10 (Uniprot Accession P10387) share 51.2% sequence identity. The Morinaga pAb recognized all groups of hordeins, notably D-hordeins, which the R5 and G12 mAb did not recognize. The Morinaga kit gave a small response on the pure oats (3.1 mg/mL), which we could not determine whether the reason was cross reaction with oat proteins, or very mild contamination during the testing process because other ELISA kits did not provide the same level of sensitivity. Put together, although the composition of wheat and barley prolamins varies as well as the antibody specificity toward each prolamin type, a more holistic detection of barley hordeins, as in Total Gluten and Morinaga, decreased the variance of wheat and barley gluten proteins rather than the detection of a specific prolamin type, as in R5 and G12 (Figure 3).

To correct the overestimation and test the ELISA kit proficiency, we introduced a common calibrator constituting total hordein isolate that allowed correcting the reporting values of all four kits, achieving satisfactory recoveries (Table 2) and reducing the variance of four kits (Figure 4B). In a real case scenario, the source of contamination is unknown. Wheat is considered as the major contamination source of gluten contamination, and all ELISA kits were designed for wheat gliadin/gluten detection, and calibrators were also from wheat. Although a special supply chain is used for gluten-free oats from farm to manufactory, barley is cultivated in the same geographic area as oats easily becoming an in-field contaminant both in Europe and Northern America.^26,44^ Rye is another possible contaminant of gluten-free oats for the same reasons; however, the cultivation of rye is less wide compared to wheat and barley.

Total hordein isolate was introduced as a common calibration in order to harmonize the kits’ results and show the variance attributed to in the extraction and antibody detection. The calculation of *z*-scores allowed for the proficiency evaluation of the ELISA kits. Better recovery was observed with the Morinaga kit at all spiking levels after calibration with the common barley hordein isolate. This may partly be due to the Morinaga extraction in an ambient-temperature overnight procedure. Better extraction efficiency was observed in the Morinaga kit than in other ELISA kits when comparing the recovery to a generic extraction method.^10^ The reason might be that the overnight ambient extraction may be more efficient to reduce disulfides of gluten proteins because the half-life of reducing agent 2-mercaptoethanol is dependent on temperature and pH. The half-life of 2-mercaptoethanol at pH 8.5 at 20 °C was 4 h, while at 40 °C, it was 1 h.^45^ Certainly, the time it takes to complete the ELISA procedure is also one factor that kit manufacturers take into consideration. Morinaga also provides an alternative short time extraction method with 10 min boiling, but we did not compare this method to other extraction methods. Raw spiked oat flour was used in this study, although another food matrix where gluten protein aggregation was induced by heat treatment may cause more severe extraction deficiency.^46^

Oat consumption as food has risen, especially in Finland, and has reached 9.4 kg per person in 2019 (5.4 kg in 2010, LUKE 2022).^47^ For people on a gluten-free diet, oats improve the nutritional quality of the diet with, for example, advantageous dietary fibers such as β-glucan, and oats have been reported to improve the quality of life in celiac disease patients.^18^ To ensure that products are safe and accurately verified as gluten-free, there needs to be a comprehensive outlook on how and where gluten proteins contaminate oats when considering the performance of gluten ELISA methods. Currently, there is no commercial assay to distinguish the detection from wheat, barley, and rye, and the use of the type I method can even lead to large discrepancy between the cereals. In this study, we showed that a comprehensive extraction method that can release all gluten proteins from a food matrix, a holistic antibody detection of all gluten protein types, and finally a calibration against a defined/certified whole gluten protein rather than a fraction of gluten can ensure reliable and accurate gluten quantification in oats.

## Supporting Information Available

(Table SI1) Step-wise spiking scheme of barley flour in pure oat flour; (Table SI2) *z*-scores of four spiking levels using four ELISA kits calibrated by the kits’ own calibration and external total hordein isolate calibration; (Figure SI3) Youden plots of *z*-scores of four levels of barley-spiked oats measured with four ELISA kits using the kits’ own calibration and total hordein isolate calibration (PDF)

## Abbreviations

2-ME: 2-mercaptoethanol
BSA: bovine serum albumin
DTT: dithiothreitol
ELISA: enzyme-linked immunosorbent assay
mAb: monoclonal antibody
pAb: polyclonal antibody
