# Supplementary material for: Quantification of Barley Contaminants in Gluten-Free Oats by Four Gluten ELISA Kits
Source: J Agric Food Chem. 2022 Feb 14;70(7):2366–73. doi: 10.1021/acs.jafc.1c07715 (PMC8880376; doi:10.1021/acs.jafc.1c07715)

Supporting Information

Quantification of barley contaminants in gluten-free oats by four gluten ELISA kits

Xin Huang[a*], Hanna Ahola[a], Matthew Daly[b], Chiara Nitride[b,c], EN Clare Mills[b], Tuula Sontag-Strohm[a]

[a]Department of Food and Nutrition, Faculty of Agriculture and Forestry, University of Helsinki, FI-00014, Helsinki, Finland.

[b]Manchester Institute of Biotechnology, Division of Infection, Immunity and Respiratory Medicine, Faculty of Biology, Medicine and Health, University of Manchester, Manchester, M1 7DN, UK.

[c]Department of Agricultural Sciences, University of Naples Federico II, 80055 Portici, Italy

*Corresponding author: xin.huang@helsinki.fi

Table SI1. Step-wise spiking scheme of barley flour in pure oat flour. Protein content of barley flour was 9.92% (N × 5.7), and total hordein content was 60.6% (based on HPLC results).

Step	1	2	3	4	5	6
Barley flour (g)	1.33					
Pure oat flour (g)	8.67	9.0	90	50	60	37.5
Take from step (g)		1.0 (step 1)	10 (step 2)	50 (step 3)	40 (step 4)	12.5
Hordein content (mg/kg)	8000	800	80	40	16	4

Table SI2. The z-scores of four spiking levels by four ELISA kits calibrated by kits' own calibration and external total hordein isolate calibration. The z-score was obtained using the formula z = (x-X)/σ, where x was the measured/calibrated content, X was the theoretical spiking content, and σ was 25% of the theoretical spiking content.

Spiking level and calibration	Replicate 1	Replicate 2	Replicate 3	Average	SD
4 mg/kg, kit standard					
R5	30.165	10.834	20.509	20.503	9.665
G12	11.889	15.219	12.354	13.154	1.803
Total Gluten	3.692	3.645	1.504	2.947	1.250
Morinaga	-0.431	0.286	2.985	0.947	1.801
4 mg/kg, hordein standard					
R5	0.998	-1.658	0.022	-0.213	1.343
G12	-1.989	-1.641	-1.939	-1.856	0.188
Total Gluten	0.449	-0.451	-0.991	-0.331	0.727
Morinaga	0.508	1.354	4.383	2.081	2.038
16 mg/kg, kit standard					
R5	19.265	15.324	13.511	16.034	2.942
G12	12.267	21.241	18.170	17.226	4.561
Total Gluten	0.481	-0.345	-0.254	-0.040	0.453
Morinaga	-0.740	-1.419	-0.057	-0.739	0.681
16 mg/kg, hordein standard					
R5	-0.693	-1.199	-1.440	-1.110	0.381
G12	-1.948	-1.065	-1.350	-1.454	0.451
Total Gluten	-1.160	-1.768	-1.703	-1.544	0.334
Morinaga	-0.062	-0.819	0.673	-0.069	0.746
40 mg/kg, kit standard					
R5	20.201	12.847	20.414	17.821	4.309
G12	18.382	16.313	16.328	17.008	1.190
Total Gluten	0.835	-0.366	0.828	0.432	0.691
Morinaga	-0.229	-1.605	-0.821	-0.885	0.690
40 mg/kg, hordein standard					
R5	-0.371	-1.435	-0.340	-0.715	0.624
G12	-1.330	-1.531	-1.529	-1.463	0.116
Total Gluten	-1.438	-0.625	-1.442	-1.168	0.471
Morinaga	0.490	-1.032	-0.151	-0.231	0.764
80 mg/kg, kit standard					
R5	14.846	15.076	17.803	15.908	1.645
G12	11.132	8.542	13.122	10.932	2.297
Total Gluten	-0.100	0.620	0.370	0.297	0.366
Morinaga	-0.920	-1.222	-1.056	-1.066	0.151
80 mg/kg, hordein standard					
R5	-1.262	-1.232	-0.878	-1.124	0.214
G12	-2.072	-2.364	-1.857	-2.097	0.254
Total Gluten	-1.557	-1.244	-1.133	-1.311	0.220
Morinaga	-0.260	-0.597	-0.411	-0.423	0.169

Figure SI3. Youden plots of z-scores of four levels of barley spiked oats (4 vs. 16 mg/kg, A and B; 16 vs. 40 mg/kg, C and D; 40 vs. 80 mg/kg, E and F) measured with four ELISA kits using the kits' own calibration (A, C and E) and total hordein isolate calibration (B, D and F). The z-score was obtained using the formula $z = (x-X)/\sigma$, where x was the measured/calibrated content, X was the theoretical spiking content, and σ was 25% of the theoretical spiking content. The black cross set at z = 0 was the theoretical spiking content and the black rectangle was set to $|z| \leq 2$, which was considered satisfactory in the proficiency assessment (ISO 13528). The blue cross was set at the mean value of all z-scores, and the blue rectangle was set at 2 times standard deviation of all the z-scores. Three points from each ELISA kit indicated three individual extraction replicates.

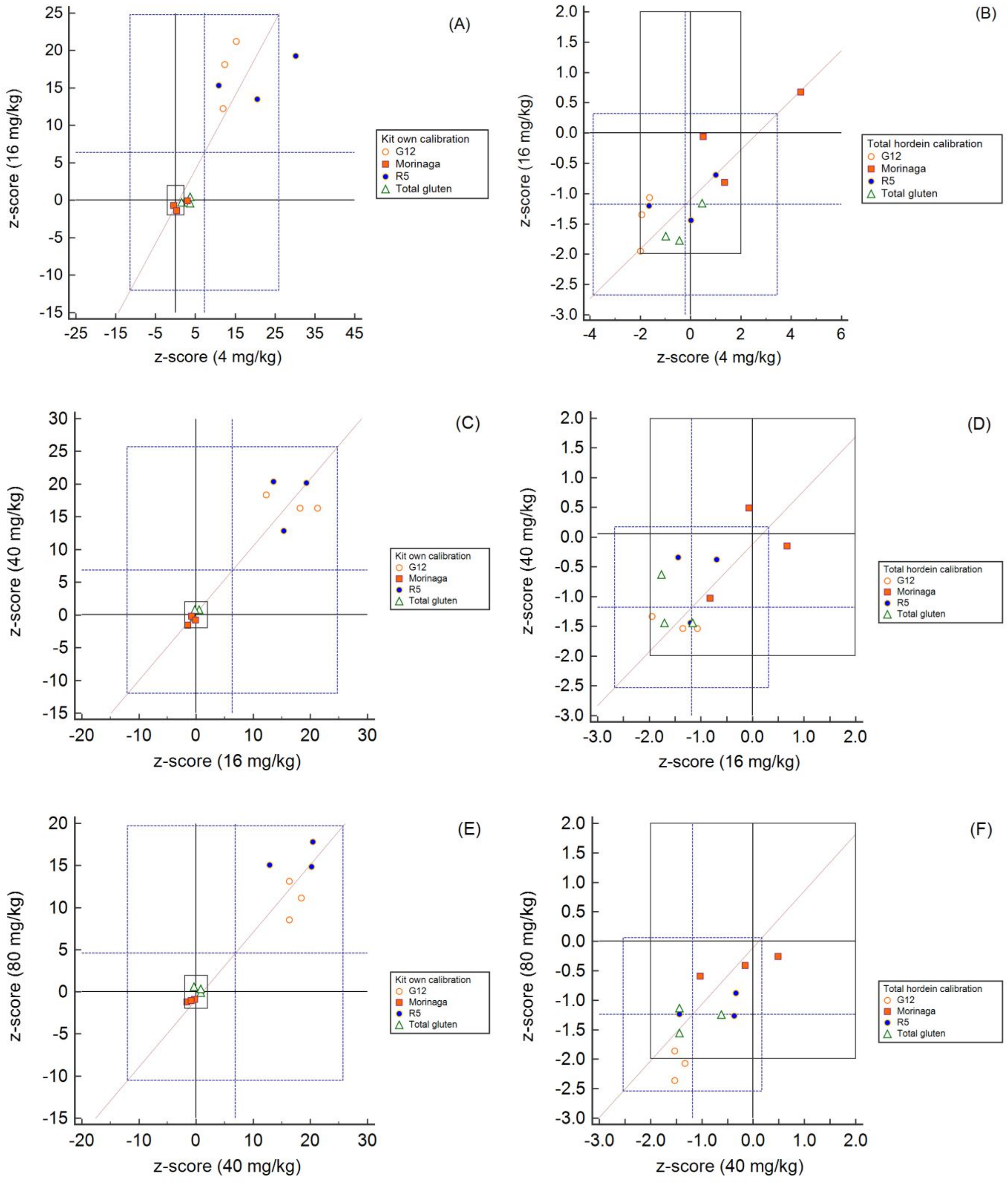

Supplement: Supplementary file 1 — jf1c07715_si_001.pdf [file jf1c07715_si_001.pdf]
